# Complete mitochondrial genome of *Echinorhynchus gadi* (Acanthocephala, Echinorhynchida) and its phylogenetic implications

**DOI:** 10.3897/zookeys.1267.177123

**Published:** 2026-01-23

**Authors:** FeiMing Chen, JinWei Gao, Yu Huang, Hao Wu, Min Xie, ZhenZhen Xiong, JiaYu Wu, Jia Cai, Rong Xu, Xiao Jin, Rui Song, DongSheng Ou

**Affiliations:** 1 Hunan Fisheries Research Institute and Aquatic Products Seed Stock Station, Changsha, Hunan, China Guangdong Ocean University Zhanjiang China; 2 Fisheries College, Guangdong Provincial Key Laboratory of Aquatic Animal Disease Control and Healthy Culture, Guangdong Ocean University, Zhanjiang, Guangdong, China Hunan Fisheries Research Institute and Aquatic Products Seed Stock Station Changsha China; 3 Key Laboratory of Biodiversity and Environment on the Qinghai-Tibetan Plateau, Ministry of Education, School of Ecology and Environment, Xizang University, Lhasa, China Xizang University Lhasa China

**Keywords:** Acanthocephala, *Echinorhynchus gadi*, mitochondrial genome, phylogenetic analysis, tRNA duplication

## Abstract

The Echinorhynchidae has a long research history, but its mitochondrial genome evolution remains poorly understood, hindering phylogenetic resolution. In this study, we report the first complete mitochondrial genome of the genus *Echinorhynchus*, obtained from its type species, *Echinorhynchus gadi*. The circular mitogenome was 17,696 bp in length and contained 39 genes: 12 protein-coding genes (lacking *atp8*), two ribosomal RNA genes, and 25 transfer RNA genes, including two extra copies of *trnW* and one extra copy of *trnV*. Five non-coding regions were identified; the major non-coding region contained tandem repeats and pseudogene fragments, consistent with a tandem duplication and random loss mechanism. Phylogenetic analysis based on the concatenated amino acid sequences of the 12 protein-coding genes placed *E. gadi* and *E. truttae* in a well-supported monophyletic clade representing the genus *Echinorhynchus*. This clade was sister to *Aspersentis megarhynchus*, supporting a close relationship between Echinorhynchidae and Heteracanthocephalidae. Because the published *E. truttae* mitogenome is incomplete, this study fills a critical genomic gap and provides a valuable molecular resource for future taxonomic, systematic, and evolutionary studies of Acanthocephala.

## Introduction

Acanthocephalans are obligate endoparasites characterized by a retractable and hook-bearing proboscis used to anchor to the intestinal wall of vertebrate hosts (Perrot-Minnot et al. 2023). This attachment can cause hemorrhage, necrosis, and inflammation, and in severe cases may even lead to intestinal perforation (Dezfuli et al. 2011). Their life cycles typically involve arthropods as intermediate hosts and vertebrates as definitive hosts, making them important in medical, veterinary, and ecological studies, as well as models for physiology and evolutionary biology (Perrot-Minnot et al. 2023; Xie et al. 2025a).

The genus *Echinorhynchus* Zoega in Müller, 1776 is a key taxon within the order Echinorhynchida, comprising at least 52 described species (Amin 2013; Wayland et al. 2015). However, its morphological homogeneity poses problems for taxonomists because relatively few anatomical characters for discriminating species (Wayland et al. 2015). Therefore, integrating morphological and molecular data is essential for reliable species delimitation. Mitochondrial DNA (mtDNA) is a useful tool due to its low recombination rate, maternal inheritance, and moderate evolutionary rate (Gao et al. 2022). Despite this, available molecular data for *Echinorhynchus* species remain limited. Only an incomplete mitochondrial genome for *Echinorhynchus truttae* Schrank, 1788 is available, lacking two protein-coding genes (PCGs) and five transfer RNAs (tRNAs), which limits its phylogenetic utility (Weber et al. 2013; Song et al. 2019; Xie et al. 2025b). Because phylogenetic analyses of acanthocephalans typically rely on all 12 PCGs, sparse and incomplete data hinder robust inference (Gao et al. 2023). Moreover, the mitochondrial genome of the type species, *Echinorhynchus gadi* Zoega in Müller, 1776, has not yet been sequenced, hindering phylogenetic reconstruction and taxonomic clarity within the genus. Previous studies suggest that *E. gadi* may actually represent a complex of morphologically cryptic species, complicating accurate species identification within the genus (Väinölä et al. 1994; Wayland et al. 2005; Wayland et al. 2015).

In this study, we sequenced and annotated the complete mitochondrial genome of *E. gadi*, providing the first complete mitochondrial genome for both the genus *Echinorhynchus* and the family Echinorhynchidae. We conducted detailed phylogenetic analyses to assess the evolutionary status of *E. gadi* within Acanthocephala. This dataset provides a foundation for species identification and future taxonomic, systematic, and evolutionary studies of Acanthocephala.

## Materials and methods

### Sample collection

Specimens of *E. gadi* were collected in May 2022 from the intestinal tracts of *Gadus chalcogrammus*, purchased at a seafood market in Dalian City, Liaoning Province, China (39°03'N, 121°52'E). Parasites were preserved in 70% ethanol for morphological examination and in 100% ethanol for molecular analyses. All specimens were stored at 4 °C. A voucher specimen (DLegadi2205) was deposited at the Hunan Fisheries Research Institute and Aquatic Products Seed Stock Station.

### DNA extraction, mitogenome sequencing, assembly, and annotation

Genomic DNA was extracted using the TIANamp Micro DNA Kit (Tiangen Biotech, Beijing, China). Primers were designed based on conserved regions of related acanthocephalan mitogenomes (Suppl. material 1). PCR amplicons were purified and Sanger-sequenced (Sangon Biotech, Shanghai) using a primer-walking strategy. Nanopore sequencing (QitanTech QPursue-6k, Aoke Biotechnology, Wuhan, China) was used to resolve repetitive regions.

Genome assembly was performed using DNASTAR v7.1 (Burland 2000), and initial annotation of PCGs, tRNAs, and ribosomal RNAs (rRNAs) was conducted with MitoZ v3.4.2 (Meng et al. 2019) and ARWEN v1.2 (Laslett and Canbäck 2008). PCG annotations were refined using NCBI ORFfinder, and gene boundaries were verified by comparison with homologous sequences. Some tRNA genes not recognized by MitoZ were identified by aligning them with published acanthocephalan tRNA sequences and were manually corrected. A circular mitogenome map was generated using Proksee (Grant et al. 2023). Codon usage and relative synonymous codon usage (RSCU) were analyzed using PhyloSuite v1.2.3 (Zhang et al. 2020).

### Phylogenetic analyses

To assess the phylogenetic status of *E. gadi*, we sequenced its complete mitochondrial genome and compared it with available acanthocephalan mitogenomes. The analysis included all available mitochondrial genomes of acanthocephalans as of 18 July 2025, including 43 taxa from across the phylum. We used concatenated amino acid sequences of 12 PCGs for phylogenetic inference, selecting *Rotaria rotatoria* and *Philodina citrina* as outgroups (Table 1). Sequences were extracted using PhyloSuite, aligned with MAFFT v7.471 (Katoh and Standley 2013), and trimmed with trimAl v1.2 (Capella-Gutiérrez et al. 2009). The optimal partitioning scheme and substitution models (Suppl. material 2) were selected using ModelFinder (Kalyaanamoorthy et al. 2017) based on the Bayesian Information Criterion. Maximum-likelihood phylogenetic analyses were performed in IQ-TREE (Nguyen et al. 2015) with 5,000 ultrafast bootstrap replicates. Bayesian inference was performed for 1 × 10^6^ MCMC generations under MrBayes v3.2 (Ronquist et al. 2012). Resulting trees were visualized using iTOL (Zhou et al. 2023).

**Table 1.** Detailed information on representatives of Acanthocephala included in the present phylogeny.

Order	Family	Species	Accession	Size	AT%	References
Bdelloidea	Philodinidae	*Rotaria rotatoria*	GQ304898	15,319	73.2	(Min and Park 2009)
		*Philodina citrina*	FR856884	14,003	77.7	(Weber et al. 2013)
Moniliformida	Moniliformidae	*Moniliformis tupaia*	OK415026	14,066	66.2	(Dai et al. 2022)
		*Moniliformis* sp.	OP413683	14,150	63.7	unpublished
Oligacanthorhynchida	Oligacanthorhynchidae	*Macracanthorhynchus hirudinaceus*	FR856886	14,282	65.2	(Weber et al. 2013)
		*Oncicola luehei*	JN710452	14,281	60.2	(Gazi et al. 2012)
Gyracanthocephala	Quadrigyridae	*Acanthogyrus cheni*	KX108947	13,695	65.3	(Song et al. 2016)
		*Acanthogyrus bilaspurensis*	MT476589	13,360	59.3	(Muhammad et al. 2023)
		*Pallisentis celatus*	JQ943583	13,855	61.5	(Pan and Nie 2013)
Neoechinorhynchida	Neoechinorhynchidae	*Neoechinorhynchus violentum*	KC415004	13,393	59.4	(Pan and Jiang 2018)
	Tenuisentidae	*Paratenuisentis ambiguus*	FR856885	13,574	66.9	(Weber et al. 2013)
Polyacanthorhynchida	Polyacanthorhynchidae	*Polyacanthorhynchus caballeroi*	KT592358	13,956	56.3	(Gazi et al. 2016)
Echinorhynchida	Echinorhynchidae	*Echinorhynchus truttae*	FR856883	13,659	63.1	(Weber et al. 2013)
		***Echinorhynchus gadi***	**PV976760**	**17,696**	**61.6**	**Present study**
	Heteracanthocephalidae	*Aspersentis megarhynchus*	PP965112	14,661	64.6	(Xie et al. 2024)
	Arhythmacanthidae	*Heterosentis pseudobagri*	OP278658	13,742	62.5	(Gao et al. 2023)
		*Heterosentis holospinus*	PQ675784	16,560	61.5	(Chen et al. 2025)
	Cavisomidae	*Cavisoma magnum*	MN562586	13,594	63.0	(Muhammad et al. 2020a)
	Pomphorhynchidae	*Pomphorhynchus bulbocolli*	JQ824371	13,915	59.9	unpublished
		*Pomphorhynchus laevis*	JQ809446	13,889	57.1	unpublished
		*Pomphorhynchus rocci*	JQ824373	13,845	60.7	unpublished
		*Pomphorhynchus tereticollis*	JQ809451	13,965	56.9	unpublished
		*Pomphorhynchus zhoushanensis*	MN602447	14,546	56.0	unpublished
		*Longicollum pagrosomi*	OR215045	14,632	55.8	(Ren et al. 2025)
	Pseudoacanthocephalidae	*Pseudoacanthocephalus* sp.	OQ588705	14,883	61.5	unpublished
		*Pseudoacanthocephalus nguyenthileae*	PP476192	13,701	56.3	(Zhao et al. 2024)
		*Pseudoacanthocephalus bufonis*	MZ958236	14,056	58.4	(Zhao et al. 2023)
	Leptorhynchoididae	*Brentisentis yangtzensis*	MK651258	13,864	68.3	(Song et al. 2019)
		*Leptorhynchoides thecatus*	AY562383	13,888	71.4	(Steinauer et al. 2005)
	Micracanthorhynchinidae	*Micracanthorhynchina dakusuiensis*	OP131911	16,309	56.8	(Gao et al. 2022)
Polymorphida	Centrorhynchidae	*Centrorhynchus clitorideus*	MT113355	15,884	55.5	(Muhammad et al. 2020c)
		*Centrorhynchus milvus*	MK922344	14,314	54.5	(Muhammad et al. 2019b)
		*Centrorhynchus aluconis*	KT592357	15,144	55.6	(Gazi et al. 2016)
		*Sphaerirostris lanceoides*	MT476588	13,478	58.0	(Muhammad et al. 2020b)
		*Sphaerirostris picae*	MK471355	15,170	58.1	(Muhammad et al. 2019a)
Polymorphida	Polymorphidae	*Southwellina hispida*	KJ869251	14,742	63.9	(Gazi et al. 2015)
		*Bolbosoma nipponicum*	OR468096	14,296	60.9	(Li et al. 2024)
		*Bolbosoma balaenae*	MZ357084	14,301	62.6	(García-Gallego et al. 2023)
		*Bolbosoma capitatum*	MZ357085	14,319	63.9	(García-Gallego et al. 2023)
		*Bolbosoma vasculosum*	MZ357087	14,313	63.9	(García-Gallego et al. 2023)
		*Bolbosoma turbinella*	MZ357086	14,199	60.4	unpublished
		*Corynosoma bullosum*	PQ516697	14,879	63.8	(Xie et al. 2025a)
		*Corynosoma evae*	PQ516696	13,947	61.6	(Xie et al. 2025a)
		*Corynosoma villosum*	OR468095	14,241	60.9	(Li et al. 2024)
		*Polymorphus minutus*	MN646175	14,149	64.4	(Sarwar et al. 2021)
	Plagiorhynchidae	*Plagiorhynchus transversus*	KT447549	15,477	61.1	(Gazi et al. 2016)

## Results

### Species identification

**Figure 1.**
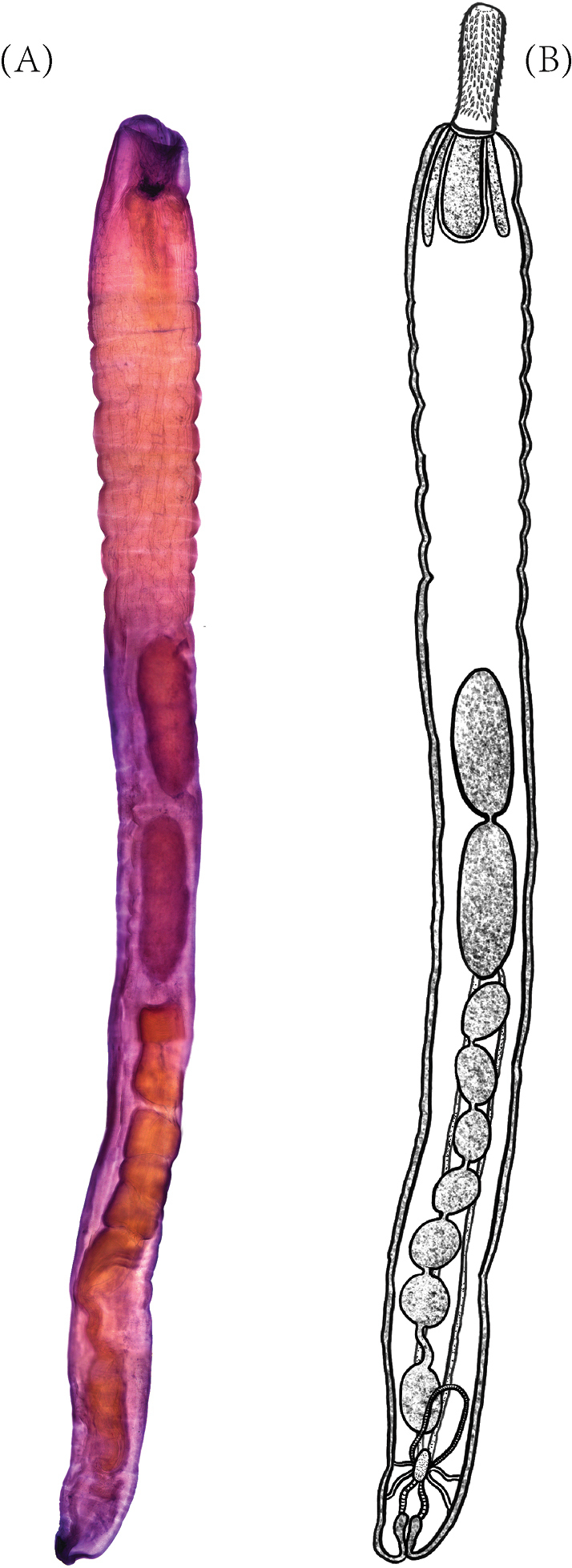
Morphology of *Echinorhynchus gadi* (**A**) and sketch (**B**).

**Figure 2.**
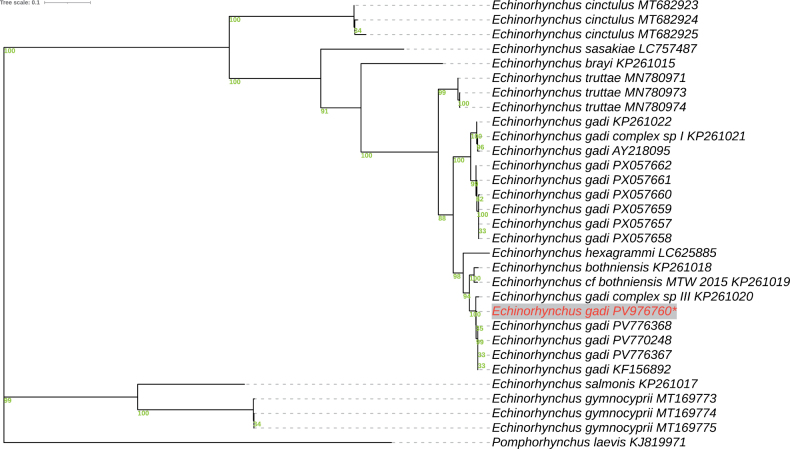
ML phylogeny of *Echinorhynchus gadi* inferred from mitochondrial cytochrome c oxidase subunit I (*cox1*) sequences. *Pomphorhynchus laevis* was used as the outgroup.

The specimens were identified as *E. gadi* based on morphological characteristics (Fig. 1), consistent with previous descriptions (Wayland et al. 2015; Amin et al. 2021). A BLASTn search of the mitochondrial *cox1* sequence showed >99% similarity to published *E. gadi* sequences, providing strong molecular support for this identification. In addition, a maximum-likelihood (ML) phylogenetic tree based on *cox1* sequences (Fig. 2) clearly placed our specimens within the *E. gadi* clade, corroborating the morphological identification.

### Mitochondrial genome organization and composition

**Figure 3.**
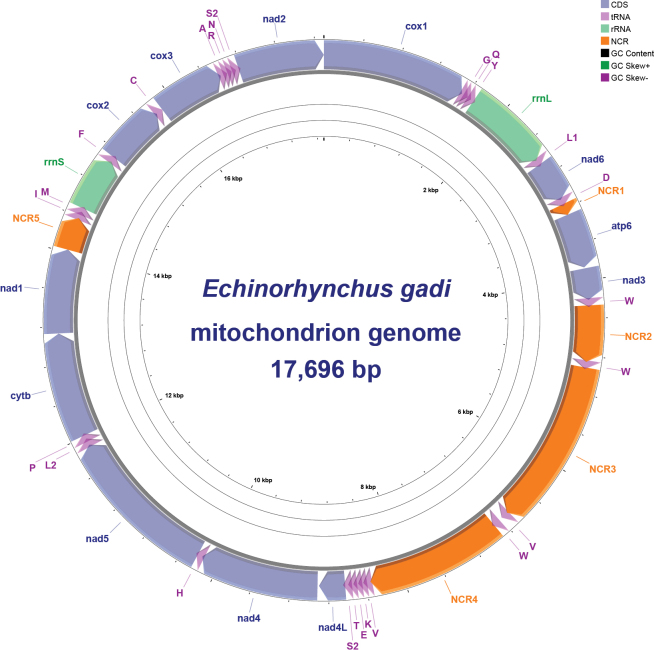
Gene map of the mitochondrial genome of *Echinorhynchus gadi*. The outermost ring depicts GC content and the innermost ring shows GC skew.

The complete mitochondrial genome of *E. gadi* was 17,696 bp in length and contained 39 genes—12 PCGs (lacking *atp8*), 2 rRNAs, and 25 tRNAs. This gene content included two extra copies of *trnW* and one extra copy of *trnV*. All genes were encoded on the heavy strand and shared the same transcriptional orientation. The genome contained five non-coding regions (NCRs) (Table 2). Nucleotide composition was A = 21.8%, T = 39.8%, C = 10.7%, and G = 27.7% (A+T = 61.6%; G+C = 38.4%). The AT-skew and GC-skew were −0.292 and 0.445, respectively. A graphical circular map of the mitogenome is shown in Fig. 3, presenting gene order and orientation.

**Table 2.** Annotations and gene organization of *Echinorhynchus gadi*.

Gene	Start	End	Size (bp)	Intergenic nucleotides	Codon start	Codon stop	Anti-codon	Strand
*cox1*	1	1539	1539		GTG	TAA		H
*trnG*	1539	1591	53	−1			TCC	H
*trnQ*	1582	1633	52	−10			TTG	H
*trnY*	1634	1686	53				GTA	H
*rrnL*	1687	2608	922					H
*trnL1*	2609	2662	54				TAG	H
*nad6*	2663	3098	436		GTG	T		H
*trnD*	3099	3151	53				GTC	H
*atp6*	3274	3858	585	122	GTG	TAA		H
*nad3*	3872	4205	334	13	ATT	T		H
*trnW*	4206	4266	61				TCA	H
*trnW–2*	4867	4927	61	600			CCA	H
*trnV*	6707	6766	60	1779			TAC	H
*trnW–3*	6836	6896	61	69			TCA	H
*trnV–2*	8353	8412	60	1456			TAC	H
*trnK*	8407	8467	61	−6			CTT	H
*trnE*	8462	8515	54	−6			TTC	H
*trnT*	8517	8571	55	1			TGT	H
*trnS2*	8572	8624	53				TGA	H
*nad4L*	8625	8900	276		ATG	TAG		H
*nad4*	8918	10186	1269	17	ATG	TAG		H
*trnH*	10187	10237	51				GTG	H
*nad5*	10241	11899	1659	3	ATG	TAG		H
*trnL2*	11899	11951	53	−1			TAA	H
*trnP*	11960	12012	53	8			TGG	H
*cytb*	12013	13131	1119		ATG	TAG		H
*nad1*	13135	14031	897	3	TTG	TAA		H
*trnI*	14385	14438	54	353			GAT	H
*trnM*	14442	14498	57	3			CAT	H
*rrnS*	14499	15078	580					H
*trnF*	15079	15131	53				GAA	H
*cox2*	15132	15780	649		ATG	T		H
*trnC*	15781	15832	52				GCA	H
*cox3*	15855	16571	717	22	ATG	TAA		H
*trnA*	16570	16622	53	−2			TGC	H
*trnR*	16622	16677	56	−1			ACG	H
*trnN*	16669	16723	55	−9			GTT	
*trnS1*	16722	16776	55	−2			ACT	
*nad2*	16777	17694	918		GTG	TAA		

### PCGs and codon usage

**Figure 4.**
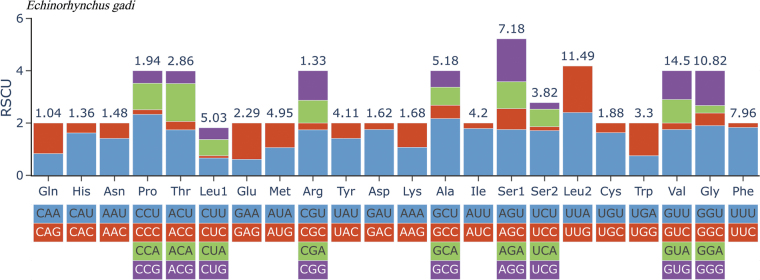
Relative synonymous codon usage (RSCU) of *Echinorhynchus gadi*. The codon families (in alphabetical order) are labelled on the x-axis. Values at the top of each bar represent amino acid usage as a percentage.

The 12 PCGs had a total length of 10,395 bp (excluding termination codons) and encoded 3,465 amino acids. Gene lengths ranged from 276 bp (*nad4L*) to 1,659 bp (*nad5*). Four PCGs started with GTG (*cox1*, *nad6*, *atp6*, *nad2*), six started with ATG (*nad4L*, *nad4*, *nad5*, *cytb*, *cox2*, *cox3*), one started with ATT (*nad3*), and one used the less common start codon TTG (*nad1*). Five PCGs terminated with TAA (*cox1*, *atp6*, *nad1*, *cox3*, *nad2*), four terminated with TAG (*nad4L*, *nad4*, *nad5*, *cytb*), and three had an incomplete stop codon T (*nad6*, *nad3*, *cox2*) (Table 2). Analysis of amino acid frequencies revealed that Val (valine) was the most prevalent amino acid, whereas Glu (glutamic acid) was the least prevalent. Accordingly, the most frequently used codons were UUU (Phe), followed by UUA (Leu) and GUU (Val), whereas the rarest codons were CCC (Pro) and CGC (Arg). Codon usage patterns are summarized in Fig. 4 and Table 3.

**Table 3.** Genetic code and codon usage for 12 PCGs in the mitochondrial genome of *Echinorhynchus gadi*.

**Codon**	**Count**	**RSCU**	**Codon**	**Count**	**RSCU**	**Codon**	**Count**	**RSCU**	**Codon**	**Count**	**RSCU**
UUU(F)	252	1.83	UCU(S)	81	1.71	UAU(Y)	100	1.41	UGU(C)	53	1.63
UUC(F)	23	0.17	UCC(S)	7	0.15	UAC(Y)	42	0.59	UGC(C)	12	0.37
UUA(L)	228	2.4	UCA(S)	32	0.67	UAA(*)	5	1.11	UGA(W)	43	0.75
UUG(L)	169	1.78	UCG(S)	12	0.25	UAG(*)	4	0.89	UGG(W)	71	1.25
CUU(L)	63	0.66	CCU(P)	39	2.33	CAU(H)	38	1.62	CGU(R)	20	1.74
CUC(L)	9	0.09	CCC(P)	3	0.18	CAC(H)	9	0.38	CGC(R)	3	0.26
CUA(L)	59	0.62	CCA(P)	17	1.01	CAA(Q)	15	0.83	CGA(R)	10	0.87
CUG(L)	43	0.45	CCG(P)	8	0.48	CAG(Q)	21	1.17	CGG(R)	13	1.13
AUU(I)	130	1.79	ACU(T)	43	1.74	AAU(N)	36	1.41	AGU(S)	83	1.75
AUC(I)	15	0.21	ACC(T)	8	0.32	AAC(N)	15	0.59	AGC(S)	38	0.8
AUA(M)	91	1.06	ACA(T)	36	1.45	AAA(K)	31	1.07	AGA(S)	49	1.03
AUG(M)	80	0.94	ACG(T)	12	0.48	AAG(K)	27	0.93	AGG(S)	78	1.64
GUU(V)	219	1.75	GCU(A)	97	2.17	GAU(D)	49	1.75	GGU(G)	178	1.9
GUC(V)	31	0.25	GCC(A)	23	0.51	GAC(D)	7	0.25	GGC(G)	45	0.48
GUA(V)	113	0.9	GCA(A)	31	0.69	GAA(E)	24	0.61	GGA(G)	27	0.29
GUG(V)	138	1.1	GCG(A)	28	0.63	GAG(E)	55	1.39	GGG(G)	124	1.33

### tRNA genes and rRNA genes

The *E. gadi* mitogenome contained 25 tRNA genes, ranging from 51 bp (*trnH*) to 61 bp (*trnW*) in length and totaling 1,383 bp (Table 2). This set included the standard 22 tRNAs plus three extra tRNA copies—two extra *trnW* and one extra *trnV*. The two *trnV* copies were identical, whereas the three *trnW* copies showed nucleotide substitutions and carried distinct anticodons: one copy had CCA and two copies had TCA (both encoding *trnW*). These differences suggested that *trnW* may be undergoing functional divergence or pseudogenization.

*rrnL* (922 bp) was located between *trnY* and *trnL1*, and *rrnS* (580 bp) was located between *trnM* and *trnF*. Their A+T contents were 65.6% and 63.1%, respectively, consistent with the overall AT-rich composition of the genome.

### Non-coding regions (NCRs)

Five NCRs were identified in the *E. gadi* mitogenome, totalling approximately 4,310 bp (≈24% of the genome) and ranging from 122 to 1,779 bp. A large tandem repeat array located between *nad3* and *trnK* comprised three units (661 bp, 1,969 bp, and 1,596 bp), each with sharply defined boundaries and high sequence similarity to the others. This array contained the duplicated *trnW* and *trnV* genes, together with a truncated *trnK* pseudogene. This pattern was consistent with an origin via tandem duplication followed by random loss.

### Phylogenetic analysis

**Figure 5.**
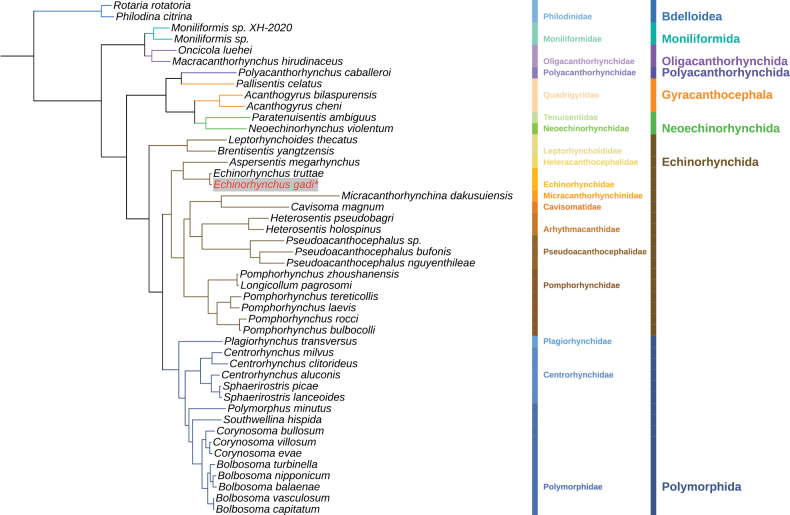
Phylogenetic analyses inferred from the ML based on concatenating amino acid sequences of 12 mitochondrial PCGs. *Rotaria rotatoria* and *Philodina citrina* are the outgroup. *Echinorhynchus gadi* is highlighted in red.

Based on the concatenated amino acid sequences of the 12 PCGs, phylogenetic analyses supported the division of Acanthocephala into three major monophyletic clades, consistent with previous studies (Fig. 5). Clade I comprised four species of Archiacanthocephala. Clade II comprised five species of Eoacanthocephala and *Polyacanthorhynchus caballeroi*. Clade III, the largest of the three clades, corresponded to Palaeacanthocephala (33 species).

Within Clade III, the analysis indicated that Echinorhynchida was polyphyletic. The Leptorhynchoididae formed an independent lineage, separated from other families of Echinorhynchida. This suggested that Leptorhynchoididae represented a distinct evolutionary lineage within the order. Furthermore, Echinorhynchidae and Heteracanthocephalidae diverged early within the main Echinorhynchida clade, supporting the hypothesis that they evolved from a common ancestor at an early stage.

Focusing on the genus *Echinorhynchus*, *E. gadi* and *E. truttae* formed a well-supported monophyletic clade. This confirmed the validity of their traditional taxonomic status within the *Echinorhynchus* and Echinorhynchidae. This clade was sister to *Aspersentis megarhynchus* (Heteracanthocephalidae), indicating a close relationship between Echinorhynchidae and Heteracanthocephalidae.

The BI analysis produced a tree topology that was congruent with the ML tree, providing additional confidence in our results (Suppl. material 3). The consistency between the ML and BI tree topologies lent additional robustness to our phylogenetic conclusions.

## Discussion

**Figure 6.**
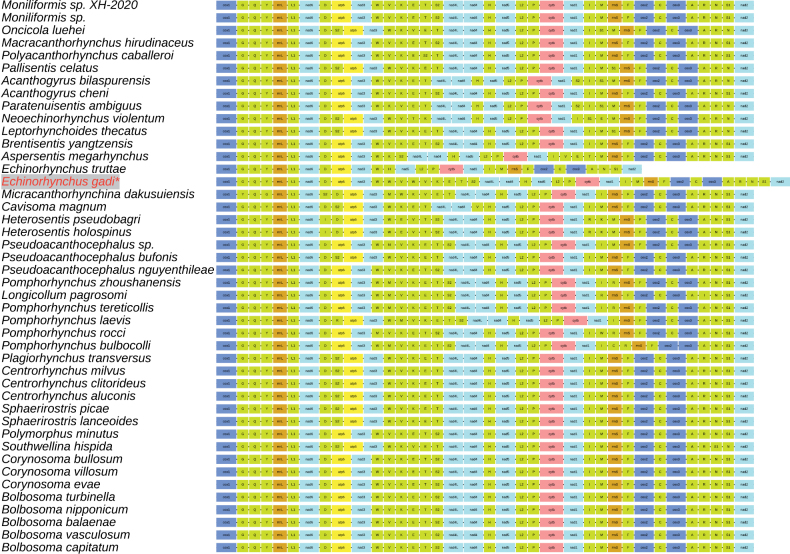
Comparison of linearized mitochondrial genome arrangement of *Echinorhynchus gadi* and other Acanthocephalan species.

The mitochondrial genome of *E. gadi* was exceptionally large for an acanthocephalan, primarily due to expanded NCRs, notably the large tandem repeat array described above (Xie et al. 2024, 2025b). These findings support the hypothesis that mitogenome size evolution in acanthocephalans is driven mainly by the accumulation of repetitive elements rather than increased coding capacity. The presence of duplicated tRNA genes within the tandem repeat array was notable. Although tandem repeats in NCRs are common in acanthocephalans, arrays that include tRNA genes have been reported only in *Leptorhynchoides thecatus* (Pan and Nie 2013; Gao et al. 2022; Chen et al. 2025). Our results provide empirical support for the “tandem duplication followed by random loss” model of mitochondrial genome evolution. This process can generate repeat arrays and sporadic gene duplications, and it has been invoked to explain gene rearrangements in animal mitogenomes. The duplicated tRNAs may contribute to functional diversification via novel anticodons, potentially enhancing translational efficiency or representing a step toward neofunctionalization (Romanova et al. 2020). Excluding the duplicated tRNAs in *E. gadi*, we found that the gene order was identical to those reported for *Bolbosoma*, *Neoechinorhynchus*, *Pseudoacanthocephalus*, and *Moniliformis* spp., indicating strong conservation of gene arrangement (Fig. 6) (Pan and Jiang 2018; Dai et al. 2022; Zhao et al. 2023, 2024; Li et al. 2024).

Phylogenetic analyses supported a close relationship between Echinorhynchidae and Heteracanthocephalidae, consistent with previous studies (Xie et al. 2024). *Echinorhynchus gadi* grouped with *E. truttae*, highlighting their close relationship, which aligns with their traditional taxonomy. Because only two *Echinorhynchus* species were included, we did not test the monophyly of the genus. Phylogenetic analyses indicated that including the incomplete *E. truttae* mitogenome did not alter the overall topology. Nevertheless, denser sampling across *Echinorhynchus* and additional complete mitogenomes will be necessary to test genus-level monophyly and to clarify interspecific relationships.

## Conclusions

We obtained the first complete mitochondrial genome of the genus *Echinorhynchus* from its type species, *E. gadi*. The *E. gadi* mitogenome was unusually large due to expanded NCRs, whereas core gene content and gene order remained highly conserved aside from tRNA duplications. Phylogenetic analyses supported a sister relationship between Echinorhynchidae and Heteracanthocephalidae. This genomic resource clarified phylogenetic relationships within the order Echinorhynchida and provided a robust molecular framework for future taxonomic, systematic, and evolutionary studies.

## Additional information

### Use of AI

No use of AI was reported.

### Author ORCIDs

FeiMing Chen https://orcid.org/0009-0000-0743-4096

JinWei Gao https://orcid.org/0000-0003-0551-1339

Yu Huang https://orcid.org/0000-0001-7589-0974

Hao Wu https://orcid.org/0000-0002-3104-4474

Min Xie https://orcid.org/0000-0003-2304-3954

ZhenZhen Xiong https://orcid.org/0009-0004-2149-2603

JiaYu Wu https://orcid.org/0009-0001-5521-1556

Jia Cai https://orcid.org/0000-0002-9501-7336

Xiao Jin https://orcid.org/0000-0002-2521-6591

Rui Song https://orcid.org/0000-0002-3856-4069

### Data availability

The complete mitochondrial genome of *E. gadi* has been deposited in the GenBank under accession number PV976760.

## Supplementary materials

Supplementary material 1 Primers and PCR gel plot of *Echinorhynchus gadi* Data type docx https://binary.pensoft.net/file/1518977This dataset is made available under the Open Database License (http://opendatacommons.org/licenses/odbl/1.0/). The Open Database License (ODbL) is a license agreement intended to allow users to freely share, modify, and use this Dataset while maintaining this same freedom for others, provided that the original source and author(s) are credited. FeiMing Chen, JinWei Gao, Yu Huang, Hao Wu, Min Xie, ZhenZhen Xiong, JiaYu Wu, Jia Cai, Rong Xu, Xiao Jin, Rui Song, DongSheng Ou

Supplementary material 2 Partitioning scheme and corresponding best-fit models for phylogenetic analysis Data type docx https://binary.pensoft.net/file/1518978This dataset is made available under the Open Database License (http://opendatacommons.org/licenses/odbl/1.0/). The Open Database License (ODbL) is a license agreement intended to allow users to freely share, modify, and use this Dataset while maintaining this same freedom for others, provided that the original source and author(s) are credited. FeiMing Chen, JinWei Gao, Yu Huang, Hao Wu, Min Xie, ZhenZhen Xiong, JiaYu Wu, Jia Cai, Rong Xu, Xiao Jin, Rui Song, DongSheng Ou

Supplementary material 3 Phylogenetic analyses inferred from the BI based on concatenating amino acid sequences of 12 mitochondrial PCGs Data type png Explanation note *Rotaria rotatoria* and *Philodina citrina* are the outgroup. *Echinorhynchus gadi* is highlighted in red. https://binary.pensoft.net/file/1518979This dataset is made available under the Open Database License (http://opendatacommons.org/licenses/odbl/1.0/). The Open Database License (ODbL) is a license agreement intended to allow users to freely share, modify, and use this Dataset while maintaining this same freedom for others, provided that the original source and author(s) are credited. FeiMing Chen, JinWei Gao, Yu Huang, Hao Wu, Min Xie, ZhenZhen Xiong, JiaYu Wu, Jia Cai, Rong Xu, Xiao Jin, Rui Song, DongSheng Ou
