# Supplementary material for: Complete mitochondrial genome of Echinorhynchus gadi (Acanthocephala, Echinorhynchida) and its phylogenetic implications
Source: Zookeys. 2026 Jan 23;1267:179–95. doi: 10.3897/zookeys.1267.177123 (PMC12859643; doi:10.3897/zookeys.1267.177123)
Supplement: Supplementary material 1 — Primers and PCR gel plot of Echinorhynchus gadi [file zookeys-1267-179_article-177123__-s001.docx]

**Supplementary file 1:** Primers used for amplification of the mitochondrial genome of *Echinorhynchus gadi*

| Fragment  No. | Gene or  region | Primer  name | Sequence (5’-3’) | Length  (bp) |
| --- | --- | --- | --- | --- |
| F1 | rrnS | JWF1 | CTGTGCCAGCGGCTGCGGTT | 406 |
|  |  | JWR1 | GACGGGCGATATGTACTCATG |  |
| F2 | rrnS-rrnL | JWF2 | TGATTGTCTAAGTCTAGTGG | 5161 |
|  |  | JWR2 | GTCTTTCCGTCTTTAACCTG |  |
| F3 | rrnL | JWF3 | GCAGTAAGTTGACTGTGCT | 329 |
|  |  | JWR3 | CTCAGATCACGTACCCTGC |  |
| F4 | rrnL-nad4 | JWF4 | GTTACTCAGGGGATAACAGG | 6719 |
|  |  | JWR4 | CTAGCAACAAAACAATACAC |  |
| F5 | nad4 | JWF5 | GGTGACTCCCAAAAGTGC | 238 |
|  |  | JWR5 | GCTATATGTACAACAGAAG |  |
| F6 | nad4-cytb | JWF6 | CGGCATGGATGTTTTAGTGC | 3007 |
|  |  | JWR6 | CTAACGAGATCATGTAATGC |  |
| F7 | cytb | JWF7 | GGTTATGTTTTGCCTTGGGGT | 430 |
|  |  | JWR7 | ACCACTCAGGCTTAATATG |  |
| F8 | cytb-rrnS | JWF8 | CTTGTATTGAGTAGTTGTATTTG | 2136 |
|  |  | JWR8 | GGTTAAGTATCCAGCCCAAGTC |  |

PCR gel plot


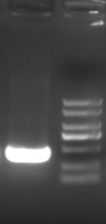


Fig.P1 **Target band fragment of JWF1R1 (*rrnS*: 406 bp)**

**DL2000 Plus DNA Marker: 2000, 1500, 1000, 750, 500, 250, 100 bp.**


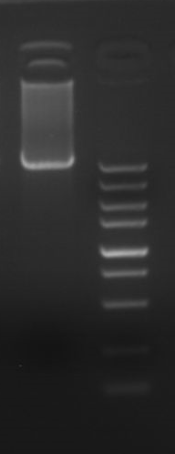


Fig.P2 **Target band fragment of JWF2R2 (*rrnS*-*rrnL*: 5161 bp)**

**DL5000 DNA Marker: 5000, 3000, 2000, 1500, 1000, 750, 500, 250, 100 bp.**


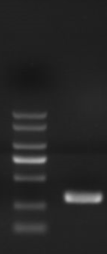


Fig.P3 **Target band fragment of JWF3R3 (*rrnL*: 329 bp)**

**DL2000 Plus DNA Marker: 2000, 1500, 1000, 750, 500, 250, 100 bp.**


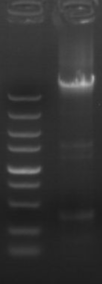


Fig.P4 **Target band fragment of JWF4R4 (*rrnL*-*nad4*: 6719 bp)**

**DL5000 DNA Marker: 5000, 3000, 2000, 1500, 1000, 750, 500, 250, 100 bp.**


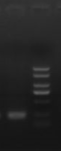


Fig.P5 **Target band fragment of JWF5R5 (*nad4*: 238 bp)**

**DL2000 Plus DNA Marker: 2000, 1500, 1000, 750, 500, 250, 100 bp.**


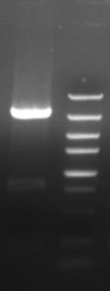


Fig.P6 **Target band fragment of JWF6R6 (*nad4*-*cytb*: 3007 bp)**

**DL5000 DNA Marker: 5000, 3000, 2000, 1500, 1000, 750, 500, 250, 100 bp.**


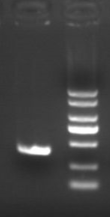


Fig.P7 **Target band fragment of JWF7R7 (*cytb*: 430 bp)**

**DL2000 Plus DNA Marker: 2000, 1500, 1000, 750, 500, 250, 100 bp.**


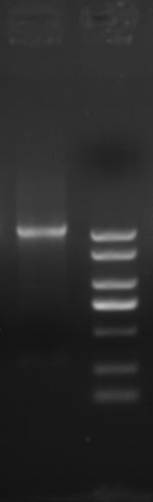


Fig.P8 **Target band fragment of JWF8R8 (*cytb*-*rrnS*: 2163 bp)**

**DL2000 Plus DNA Marker: 2000, 1500, 1000, 750, 500, 250, 100 bp.**
