# Supplementary material for: Complete mitochondrial genome of Echinorhynchus gadi (Acanthocephala, Echinorhynchida) and its phylogenetic implications
Source: Zookeys. 2026 Jan 23;1267:179–95. doi: 10.3897/zookeys.1267.177123 (PMC12859643; doi:10.3897/zookeys.1267.177123)
Supplement: Supplementary material 2 — Partitioning scheme and corresponding best-fit models for phylogenetic analysis [file zookeys-1267-179_article-177123__-s002.docx]

**Supplementary file 2**: Partitioning Scheme and Corresponding Best-Fitting Models for Phylogenetic Analysis

| Method | Subset partitions | Best model |
| --- | --- | --- |
| ML | P1: (*atp6*, *cox3*, *nad2*, *nad3*, *nad4L*, *nad4*, *nad5*, *nad6*) | MTINV+F+R6 |
|  | P2: (*cox1*, *cox2*, *cytb*, *nad1*) | MTINV+F+R5 |
|  |  |  |
| BI | P1: (*atp6*, *cox3*, *nad2*, *nad3*, *nad4L*, *nad4*, *nad5*, *nad6*) | VT+F+I+G4 |
|  | P2: (*cox1*, *cox2*, *cytb*, *nad1*) | WAG+F+I+G4 |
