# Supplementary figures and images for: Complete mitochondrial genome of Echinorhynchus gadi (Acanthocephala, Echinorhynchida) and its phylogenetic implications
Source: Zookeys. 2026 Jan 23;1267:179–95. doi: 10.3897/zookeys.1267.177123 (PMC12859643; doi:10.3897/zookeys.1267.177123)

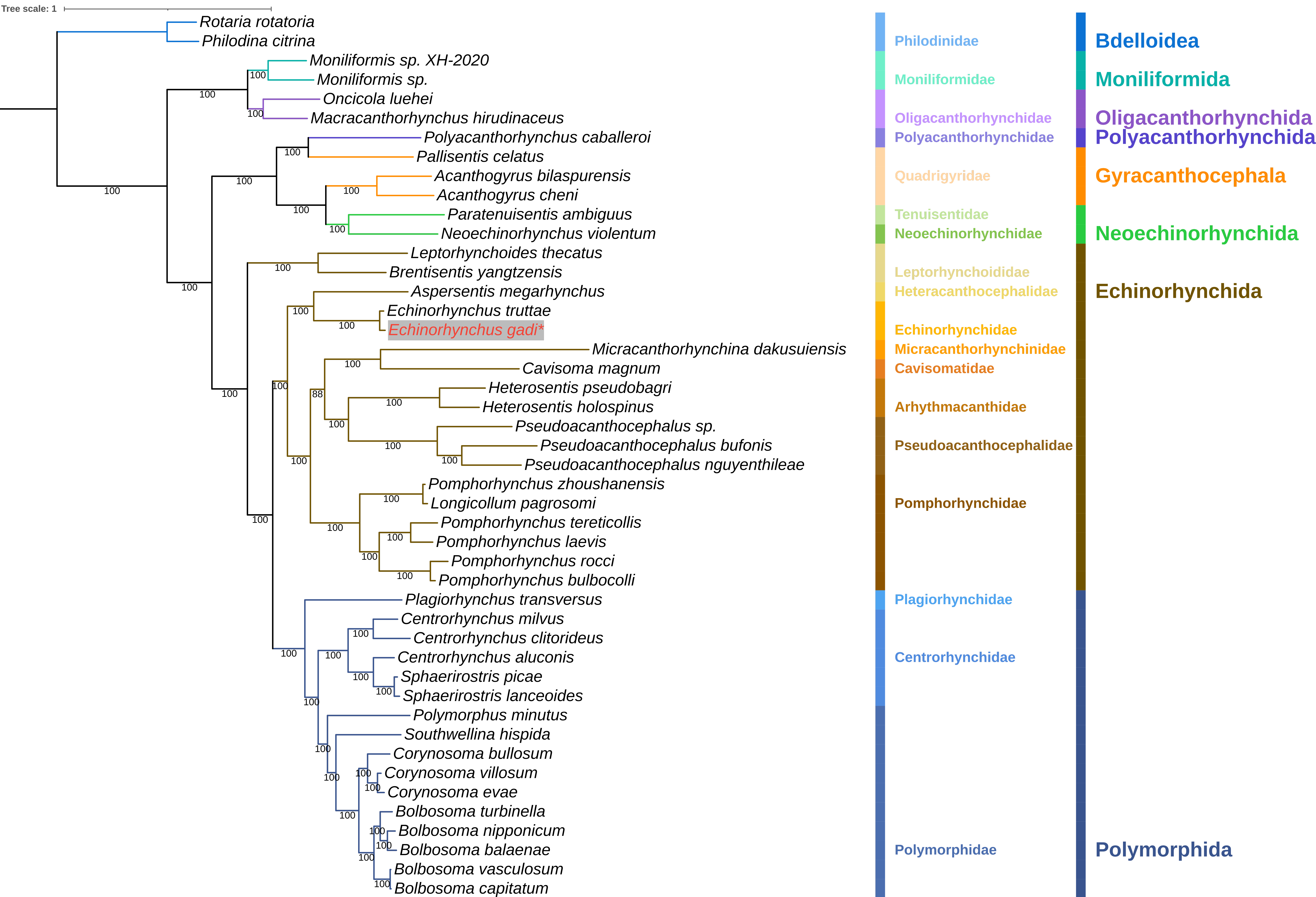

Supplement: Supplementary material 3 — Phylogenetic analyses inferred from the BI based on concatenating amino acid sequences of 12 mitochondrial PCGs [file zookeys-1267-179_article-177123__-s003.png]
